# KIAA1429 regulates lung adenocarcinoma proliferation and metastasis through the PI3K/AKT pathway by modulating ARHGAP30 expression

**DOI:** 10.1111/1759-7714.15327

**Published:** 2024-05-08

**Authors:** Wei Guo, Tan Wang, Qilin Huai, Lei Guo, Xiaobing Wang, Jie He

**Affiliations:** ^1^ Department of Thoracic Surgery, National Cancer Center/National Clinical Research Center for Cancer/Cancer Hospital Chinese Academy of Medical Sciences and Peking Union Medical College Beijing China; ^2^ Key Laboratory of Minimally Invasive Therapy Research for Lung Cancer Chinese Academy of Medical Sciences Beijing China; ^3^ State Key Laboratory of Molecular Oncology, National Cancer Center/National Clinical Research Center for Cancer/Cancer Hospital Chinese Academy of Medical Sciences and Peking Union Medical College Beijing China; ^4^ Department of Pathology, National Cancer Center/National Clinical Research Center for Cancer/Cancer Hospital Chinese Academy of Medical Sciences and Peking Union Medical College Beijing China

**Keywords:** ARHGAP30, KIAA1429, LUAD, mRNA stability, N6‐methyladenosine

## Abstract

**Background:**

Alterations in epigenetic factors are recognized as key contributors to the emergence of human cancer. The active and reversible alteration of N6‐methyladenosine (m6A) RNA is crucial for controlling gene activity and determining cellular destiny. Even with these insights, the triggering of KIAA1429 (also called VIRMA) and its role in lung adenocarcinoma (LUAD) is mostly unclear. As a result, the objective of this study was to elucidate how KIAA1429 contributes to cancer development in LUAD.

**Methods:**

This study utilized multiple methods for investigation, encompassing the in vitro functional examination of KIAA1429 in lung adenocarcinoma cells, transcriptome sequencing, methylation RNA immunoprecipitation sequencing (MeRIP‐seq), as well as RNA stability tests to ascertain the half‐life and stability of the target genes.

**Results:**

The results indicated that modifying the expression of KIAA1429 regulated the proliferation and metastasis of LUAD. By employing transcriptome sequencing alongside MeRIP‐seq analysis, the research pinpointed genes affected by m6A alterations triggered by KIAA1429. In a more detailed manner, it was discovered that KIAA1429 plays a regulatory role in the expression of ARHGAP30. Suppressing KIAA1429 results in reduced m6A levels in the mRNA of the target gene ARHGAP30, boosting its stability and expression, thus inhibiting tumor proliferation and metastasis.

**Conclusion:**

This study revealed the activation mechanism and pivotal function of KIAA1429 in LUAD tumor development, paving the way for molecular‐based interventions for LUAD.

## Graphical abstract

High expression of KIAA1429 in lung adenocarcinoma increases the m6A level of the downstream target gene ARHGAP30, which leads to a decrease in the expression of ARHGAP30, thus contributing to lung adenocarcinoma progression.

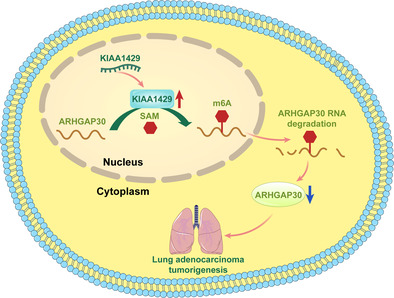

## INTRODUCTION

Non‐small cell lung cancer (NSCLC), a prevalent lung cancer variant, ranks as a primary global cause of cancer‐related fatalities.^1^ Lung adenocarcinoma (LUAD) is classified as a primary variant of NSCLC.^2^ In recent times, global instances and death rates of LUAD have escalated, primarily because of tardy detection in later phases, severely restricting clinical treatment choices.^3^ The worldwide frequency and death rates of LUAD have risen, mainly due to its delayed identification in later phases, significantly limiting the options for medical intervention.^4^

A growing body of research underscores the vital impact of genetic, epigenetic, and transcriptome alterations in the progression of LUAD.^5^ Epigenetic regulation is notably acknowledged for its crucial effect on gene activation, crucially influencing the emergence and progression of LUAD.^6^ Classic modifications in epigenetic dynamics encompass the alteration of DNA methylation and histone structure alterations.^7^ Recent studies have highlighted the critical role of RNA alterations in influencing numerous biological mechanisms, such as the development of tumors.^8^ As an example, IGF2BP3, an RNA‐binding protein, improves the steadiness and translation of EGFR mRNA, thereby boosting colorectal cancer cells' resistance to cetuximab, an EGFR‐targeting antibody. Accomplishment of this is through the activation of the EGFR pathway, reliant on N6‐methyladenosine (m6A) and in collaboration with METTL14.^9^ Research indicates that GBAP1, stimulated by METTL3, facilitates the movement, penetration, and multiplication of HCC cells through the GBAP1/miR‐22‐3p/BMPR1A/SMAD pathway, hinting at the role of GBAP1 as a possible prognostic marker and target for HCC treatment.^10^

Among the various chemical alterations found at the RNA stage, m6A RNA methylation stands out as the most common.^11^ The regulation of gene expression occurs through its impact on transcript stability, splicing, the efficiency of translation and translation independent of the cap.^12^ Comprising RNA methyltransferases METTL3, METTL14, WTAP, and KIAA1429 (also referred to as VIRMA), the m6A methyltransferase complex attaches m6A alterations to RNA. The complex forms an interaction with YTH m6A RNA‐binding proteins, namely YTHDF1, YTHDF2, and YTHDF3,^13^ which are crucial in recognizing m6A modifications. Recent evidence has linked m6A modifications to cancer proliferation, invasion and metastasis. RNA methyltransferases are crucial for m6A methylation and studies have shown that dysregulation of these enzymes plays a critical role in tumorigenesis.^14^ TROAP, which is regulated by METTL3, accelerates NSCLC progression through the PI3K/AKT and EMT pathways, highlighting TROAP as a potential novel target for NSCLC therapy.^15^

Despite these findings, the underlying mechanisms of activating m6A methyltransferases in tumor formation are mostly unclear. The aim of this study was to elucidate the reasons behind the activation of RNA methyltransferase KIAA1429 and to delve into its biological functions and molecular regulation processes in LUAD tumor formation.

## METHODS

### Datasets and database used in this study

Data on lung adenocarcinoma (LUAD) patients, including clinical specifics, from the Cancer Genome Atlas (TCGA) was acquired from the webpage of the TCGA data portal (https://portal.gdc.cancer.gov/projects/TCGA-LUAD).

Data on clinical patients in this research came from 80 LUAD patients who had R0 resections from June 2006 to June 2014. Criteria for inclusion included: extensive surgical R0 removal and LUAD verified through histology. Criteria for exclusion included: patients who underwent chemotherapy and/or radiotherapy before surgery, those without comprehensive clinical data and those who could not continue with regular follow‐ups. Every patient provided knowledgeable agreement prior to undergoing surgery. The clinical and pathological information of patients with LUAD was documented, encompassing their age, gender, tumor location, tumor differentiation status, T stage, lymph node metastasis, and TNM stage. Two pathologists pathologically verified every specimen. The pathological classification of the primary tumor and the degree of lymph node metastasis were verified based on TNM stage 8. The research adhered to the guidelines set forth in the Declaration of Helsinki. This study received approval from the Clinical Research Ethics Committee at the National Cancer Center/Chinese Academy of Medical Sciences Cancer Hospital. During the initial 2 years post‐surgery, patients underwent consistent check‐ups (every 3–6 months) in the outpatient unit, followed by yearly check‐ups. Subsequent follow‐up encompassed documenting the medical background of the patient, conducting a physical check‐up, and performing a chest computed tomography (CT) scan. The final follow‐up occurred on May 4, 2023. Table S1 enumerates the comprehensive clinical features of the 80 patients with LUAD.

### Cell culture

Cell lines A549, NCI‐H1299 and H460 were acquired from Cell Resource Center, IBMS, CAMS/PBMC. The cell lines A549, NCI‐H1299 and H460 were grown in RPMI‐1640 medium (Gibco). Every medium consisted of 10% fetal bovine serum (FBS), supplemented with penicillin (100 U/mL) and streptomycin (0.1 mg/mL). Cells underwent cultivation in a cell culture incubator maintained at 37°C in an atmosphere containing 5% CO_2_.

### Transfection

Table S2 shows the nucleotide sequences of si‐KIAA1429 and si‐ARHGAP30. Transfection agents, specifically the lipofectamine RNAiMAX kit (Invitrogen), were employed and the transfection process was executed as per the manufacturer's guidelines. Typically, tumor cells were cultivated in six‐well plates and subjected to siRNA and transfection agents the following day. The KIAA1429 knockdown lentiviral vector was acquired from Syngenbio. Thriving LUAD cells, cultivated in six‐well plates, were exposed to KIAA1429 knockdown lentivirus or control, followed by puromycin screening to achieve consistent knockdown cell lines.

### RNA isolation and quantitative reverse transcription‐ polymerase chain reaction

Cells A549, NCI‐H1299, and H460 underwent lysis using TRIzol reagent, followed by the extraction of total RNA and subsequent reverse transcription to cDNA with reverse transcription kit (Takara Bio USA, Inc.). The qRT‐PCR process was executed using TB Green (Takara Bio USA Inc.), adhering to the guidelines provided by the manufacturer. The results were adjusted in relation to glyceraldehyde‐3‐phosphate dehydrogenase (GAPDH). The primers employed in this study can be found in Table S2.

### Cell proliferation assays

Cultivation of A549 and NCI‐H1299 cells took place in 96‐well plates by incubation at 37°C in a 5% CO_2_ atmosphere for periods of 24, 48, 72, and 96 h. During the assay phase, 10 μL of cell counting kit‐8 (CCK8) was mixed into 100 μL of medium for each well, followed by a 1‐h incubation with the cells at 37°C and 5% CO_2_. Absorption levels at 450 nm were gauged using a microplate reader (Bio‐Rad).

### Colony formation assays

In every section of a six‐well culture plate, 1 × 10^3^ cells were cultivated and maintained in the culture medium for a period of 12–14 days. Following the incubation phase, the cells underwent fixation with 4% paraformaldehyde fix solution (Beyotime Biotechnology) and were then colored using 0.1% crystal violet staining solution (Beyotime Biotechnology) for a duration of 15 min. The number of colonies was analyzed to assess their capacity to form under varied experimental scenarios. Every test was conducted thrice.

### Cell migration assays

The upper chamber (Corning) was supplemented with a serum‐free culture medium, and the lower chamber received a medium with 10% FBS. A suitable quantity of cells were planted in the upper chamber. Post 24‐h incubation period, the cells traversing the membrane were fixed and colored using 4% paraformaldehyde fix solution and 0.1% crystal violet staining solution. Following this, photographs were taken with a reversed microscope (Leica). The study was conducted three times.

### Western blot assay and antibodies

Complete cellular protein was isolated from proliferating cells utilizing lysis buffer (Beyotime Biotechnology). Protein quantities were determined using enhanced BCA protein assay kit (Beyotime Biotechnology). A total of 20 μg of protein was isolated through a 10% SDS‐PAGE gel and subsequently transferred to polyvinylidene fluoride (PVDF) membranes (Millipore) via electrophoresis. The membranes underwent a triple wash using TBST and were then treated with 5% skim milk for blocking. Antibodies targeting KIAA1429, ARHGAP30, MMP2, ZEB1, β‐catenin, E‐cadherin, N‐cadherin, vimentin, p‐PI3K, PI3K, p‐AKT, AKT and GAPDH (Cell Signaling Technology; diluted 1:1000) were incubated overnight at 4°C. After being treated with primary antibodies, the membranes were subjected to a 2‐h incubation at room temperature with secondary antibodies. Visualization of protein bands was achieved using an ECL kit (ThermoFisher Scientific).

### Transcriptome sequencing

RNA extraction was carried out on both KIAA1429 knockdown and control A549 cells. By adhering to the manufacturer's instructions, total RNA was stripped of rRNAs through the use of Ribo‐Zero rRNA Removal Kits (Illumina). The RNA underwent preprocessing, and sequencing libraries were created utilizing the TruSeq Stranded Total RNA Library Prep Kit (Illumina). For managing and assessing the quality of the library, the BioAnalyzer 2100 (Agilent Technologies) was employed. Adhering to Illumina's sequencing methods, the libraries underwent denaturation into single‐stranded DNA at 10 pM, were ensnared by the Illumina flowcell, expanded on‐site into groupings, and then subjected to 150 cycle paired‐end sequencing using the Illumina NovaSeq 6000 sequencer. Gene expression variations were examined using gene ontology (GO) enrichment techniques. The corresponding data are listed in Table S3.

### Immunohistochemical staining of patient tissue chips

Tumor sections (5 μm) were dewaxed, rehydrated, and quenched. Following this, antigen retrieval was conducted utilizing citrate buffer. Following the inhibition of innate peroxidase activity using goat serum (G9023, Sigma‐Aldrich) at 37°C for an hour, the samples were incubated overnight at 4°C with anti‐ARHGAP30 antibody (A17847, ABclonal) or anti‐VIRMA/KIAA1429 antibody (ab246982, Abcam). Detection of bound antibodies was facilitated using the MaxVision HRP‐Polymer anti‐Mouse/Rabbit IHC Kit (PV‐9000, ZSGB‐BIO Corporation). Finally, staining of the sections was accomplished with diaminobenzidine (ZLI‐9019, ZSGB‐BIO Corporation), followed by counterstaining with Mosaic version 2.1 (Tucsen Photonics Co., Ltd.).

### Gene set enrichment analysis (GSEA)

GSEA was utilized to analyze molecules in the sequencing data with clusterProfiler (version 4.4.4) in the R software (version 4.2.1).

### Methylated RNA immunoprecipitation sequencing (MeRIP‐seq)

The RiboMinus Eukaryote kit version 2 (Invitrogen) was utilized for the extraction and purification of total RNA. The isolated RNA was segmented into two parts: the first part was subjected to m6A RNA immunoprecipitation (MeRIP) with the GenSeq m6A‐MeRIP Kit (GenSeq), and the second part functioned as a benchmark for standard transcriptome sequencing library assembly. Following this, RNA sequencing libraries were created using the NEBNext Ultra II Directional RNA Library Prep Kit, utilizing both the input RNA samples (which were not processed through immunoprecipitation) and the IP RNA samples (which underwent immunoprecipitation). The quality of the library was evaluated through the utilization of the BioAnalyzer 2100 device (Agilent). The Illumina NovaSeq 6000 sequencer was used for high‐throughput sequencing, operating in a 150 bp paired‐end mode. Following the analysis of images, identification of bases, and quality assurance, unprocessed data (raw reads) were produced. The preliminary quality assurance process utilized Q30 filtering. Following this, adapters and reads of inferior quality were eliminated using the cutadapt software (version 1.9.3), leading to the creation of high‐quality clean reads. Subsequently, the Hisat2 software (version 2.0.4) was utilized to synchronize all pristine reads with the human reference genome (HG19). Methylated genes in each specimen were pinpointed using MACS software, and subsequently, differential methylation gene identification was conducted through diffReps software. A specialized software was employed to sift through peaks found on mRNA exons and to execute related annotations.

### RNA stability assays

A549 and NCI‐H1299 cells were subjected to a 24h transfection procedure employing si‐NC and si‐KIAA1429. Post‐transfection, the cells underwent treatment with 10 μg/mL of actinomycin D (Sigma Aldrich). The cells underwent total RNA extraction at 0, 4, and 8h post‐treatment for subsequent examination. The RNA extraction was conducted using qRT‐PCR, with each test being replicated thrice.

### Statistical analysis

GraphPad Prism 8 served as the tool for creating graphs and conducting statistical evaluations. The determination of statistical relevance among groups was conducted through the application of unpaired student's *t*‐tests. The analysis of various conditions utilized either one‐way or two‐way ANOVAs. Results are represented as mean ± standard deviation. The criteria for statistical significance were set as: *p < 0.05, **p < 0.01; ***p < 0.001, ****p < 0.0001.

## RESULTS

### KIAA1429 is highly expressed in lung adenocarcinoma and is associated with poor prognosis

**FIGURE 1:**
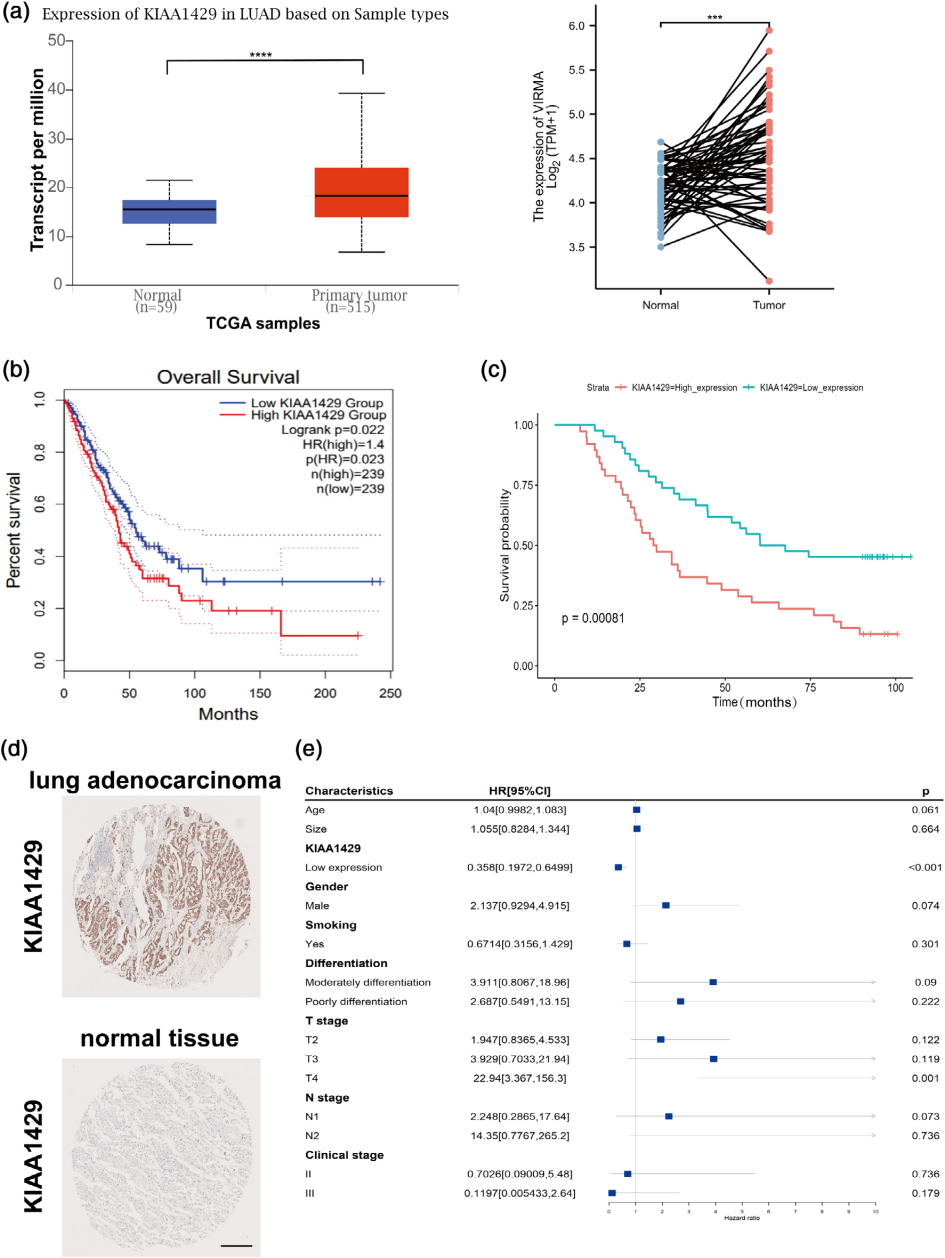
KIAA1429 is highly expressed in lung adenocarcinoma (LUAD) and associated with poor prognosis. (a) Analyzing the expression levels of KIAA1429 in lung carcinomatous tissues versus neighboring ones through TCGA paired samples. (b) Analyzing TCGA LUAD data revealed that elevated KIAA1429 expression, in contrast to lower‐level patients, suggested a lower survival rate. (c) Analysis of LUAD data from the National Cancer Center revealed that elevated KIAA1429 expression was negatively associated with patient prognosis (analyzing 80 LUAD sample patients with comprehensive prognostic data). (d) Immunohistochemistry of the KIAA1429 gene in LUADs and normal tissue. Scale bar, 500 μm. (e) Univariate meta‐regression for different subgroup analyses. Data are shown as the mean ± SD. *p < 0.05; **p < 0.01; ***p < 0.001; ****p < 0.0001, *ns* not significant.

The study showed a significant overexpression of KIAA1429 in lung adenocarcinoma tissues relative to surrounding normal tissues, as indicated by information from the TCGA database (Figure 1a). Additionally, elevated KIAA1429 levels were significantly associated with poorer patient prognosis within the TCGA dataset (*p* < 0.05) (Figure 1b). Advanced investigation using LUAD datasets from the National Cancer Center revealed that patients exhibiting high KIAA1429 levels experienced significantly lower survival rates than those with lower expression levels (Figure 1c). Immunohistochemical assays on tumor samples further corroborated the high expression levels of KIAA1429 (Figure 1d). These findings collectively suggest that KIAA1429 upregulation is prevalent in tumor tissues compared to normal tissues and is correlated with an adverse prognostic outcome. In the clinical cohort (Figure 1e), a hazard ratio (HR) value of 0.358 (*p* < 0.001) for the low KIAA1429 expression group underscores its statistical significance and high expression in LUAD patients. Moreover, the HR for the T4 stage in the T stage was 22.94, implying a 77.06% reduced risk of recurrence with low KIAA1429 expression at the T4 stage in comparison to high expression. The absence of overlap with the null line in the survival analysis graph further indicates a statistically significant difference.

### KIAA1429 regulates the proliferation and migration of LUAD cells in vitro

**FIGURE 2:**
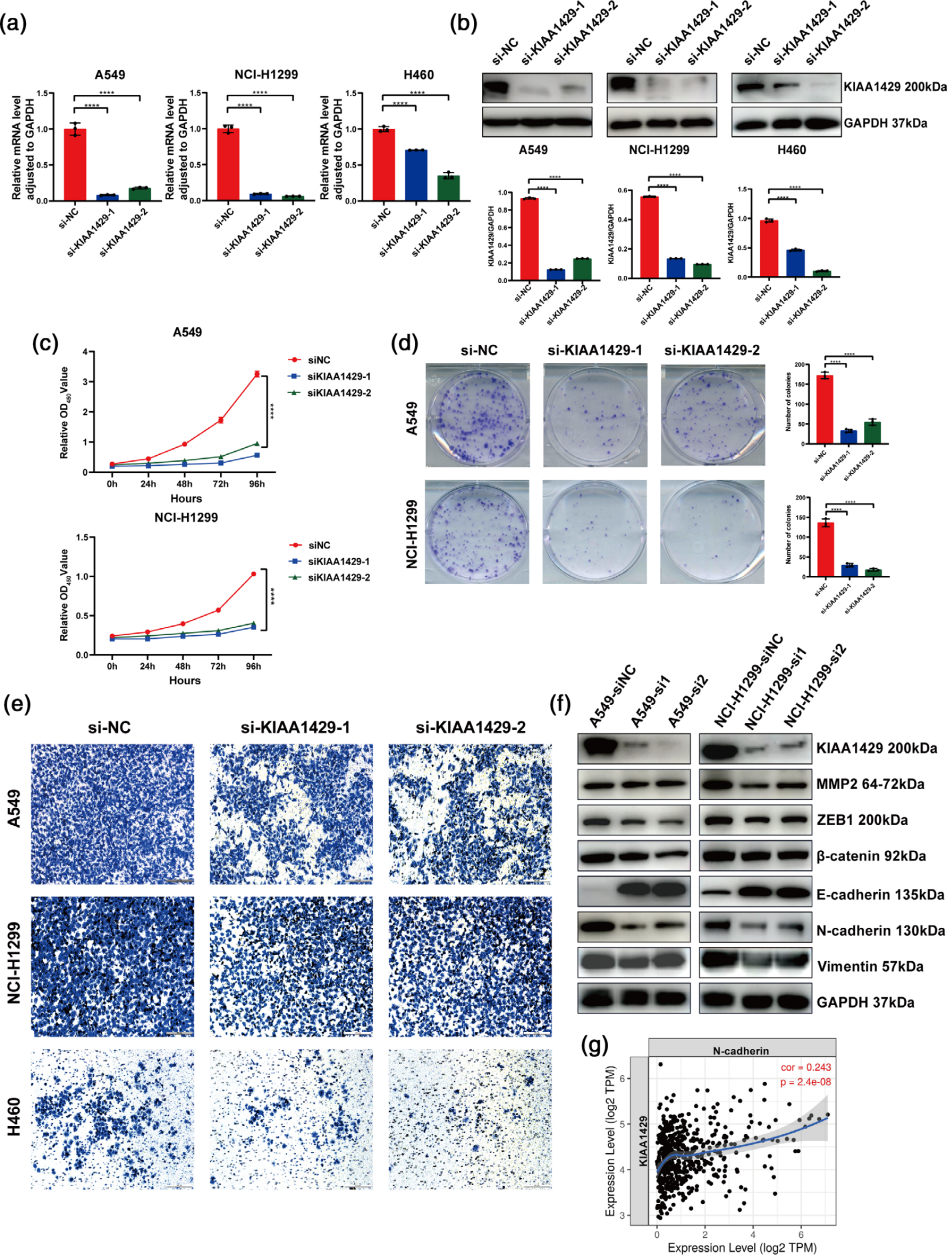
KIAA1429 regulates the proliferation and migration of lung adenocarcinoma (LUAD) cells in vitro. (a) The expression levels of KIAA1429 in A549, NCI‐H1299 and H460 cells after transfection with siNC and siKIAA1429 were compared using quantitative reverse transcription‐polymerase chain reaction (qRT‐PCR). (b) Following transfection of A549, NCI‐H1299, and H460 cells with siNC and siKIAA1429, western blotting was used to detect KIAA1429 expression. (c) The viability of A549 and NCI‐H1299 cells was assessed using cell counting kit‐8 (CCK8) assays following the silencing of KIAA1429, in comparison to the control groups. (d) Following the suppression of KIAA1429, the capacity of A549 and NCI‐H1299 cells to form colonies was identified. (e) Transwell assays were used to examine the migratory ability of A549, NCI‐H1299, and H460 cells after transfection with sh‐KIAA1429 plasmid. Scale bar, 200 μm. (f) After transfection with sh‐KIAA1429 plasmid in A549 and NCI‐H1299 cells, the expressions of KIAA1429, MMP2, ZEB1, β‐catenin, E‐cadherin, N‐cadherin, vimentin and glyceraldehyde‐3‐phosphate dehydrogenase (GAPDH) were detected by western blotting. (g) An analysis of correlations in LUAD tissues, sourced from TCGA databases, revealed a direct positive link between KIAA1429 and N‐cadherin. Three independent experiments were performed. Data are shown as the mean ± SD. *p < 0.05; **p < 0.01; ***p < 0.001; ****p < 0.0001, *ns* not significant.

Our research into the biological role of KIAA1429 in LUAD involved conducting experiments on three specific LUAD cell lines: A549, NCI‐H1299 and H460. Given the elevated expression levels of KIAA1429 in these cells, we employed siRNA technology to reduce its expression. The effectiveness of the knockdown was confirmed at the mRNA and protein levels, as depicted in Figure 2a,b. Subsequent CCK‐8 assay demonstrated that KIAA1429 suppression significantly curtailed cell proliferation (Figure 2c). Moreover, the ability of cells to form clones was notably diminished following KIAA1429 knockdown, as evidenced by the clone formation assay (Figure 2d).

Further supporting these findings, the transwell assay indicated reduced cell migration post‐KIAA1429 knockdown (Figure 2e). The study also explored the impact of KIAA1429 knockdown on proteins indicative of the epithelial‐mesenchymal transition (EMT) phenotype in tumors. Western blotting assays showed increased E‐cadherin expression after KIAA1429 knockdown, signifying a reversion to the epithelial phenotype in tumor cells. Simultaneously, there was a downregulation of mesenchymal markers (vimentin, N‐cadherin, MMP2, ZEB1), suggesting a diminished mesenchymal phenotype (Figure 2f). Additionally, we employed the TIMER2.0 database to assess the correlation between KIAA1429 and EMT‐related genes, revealing a positive correlation between KIAA1429 and N‐cadherin at the mRNA level (Figure 2g). Collectively, our data indicate that KIAA1429 silencing leads to reduced migratory and EMT phenotypes in tumor cells, thus underscoring the oncogenic role of KIAA1429 in LUAD progression.

### KIAA1429 affected the overall m6A modification level

To better grasp the significance of KIAA1429 and its subsequent targets in LUAD, RNA transcriptome sequencing and MeRIP‐seq were employed to examine the altered gene expression following the KIAA1429 suppression. In A549 cells with knockdown of KIAA1429, transcriptome sequencing identified two distinct sets of differentially expressed genes when applying a fold change (FC) greater than 3 as the threshold. Specifically, 3610 and 2636 genes showed differential expression in the KA‐SH1 and KA‐SH2 groups, respectively. Among these genes, 1467 genes followed the same directional trend, of which 892 genes were upregulated and 575 genes were downregulated.

**FIGURE 3:**
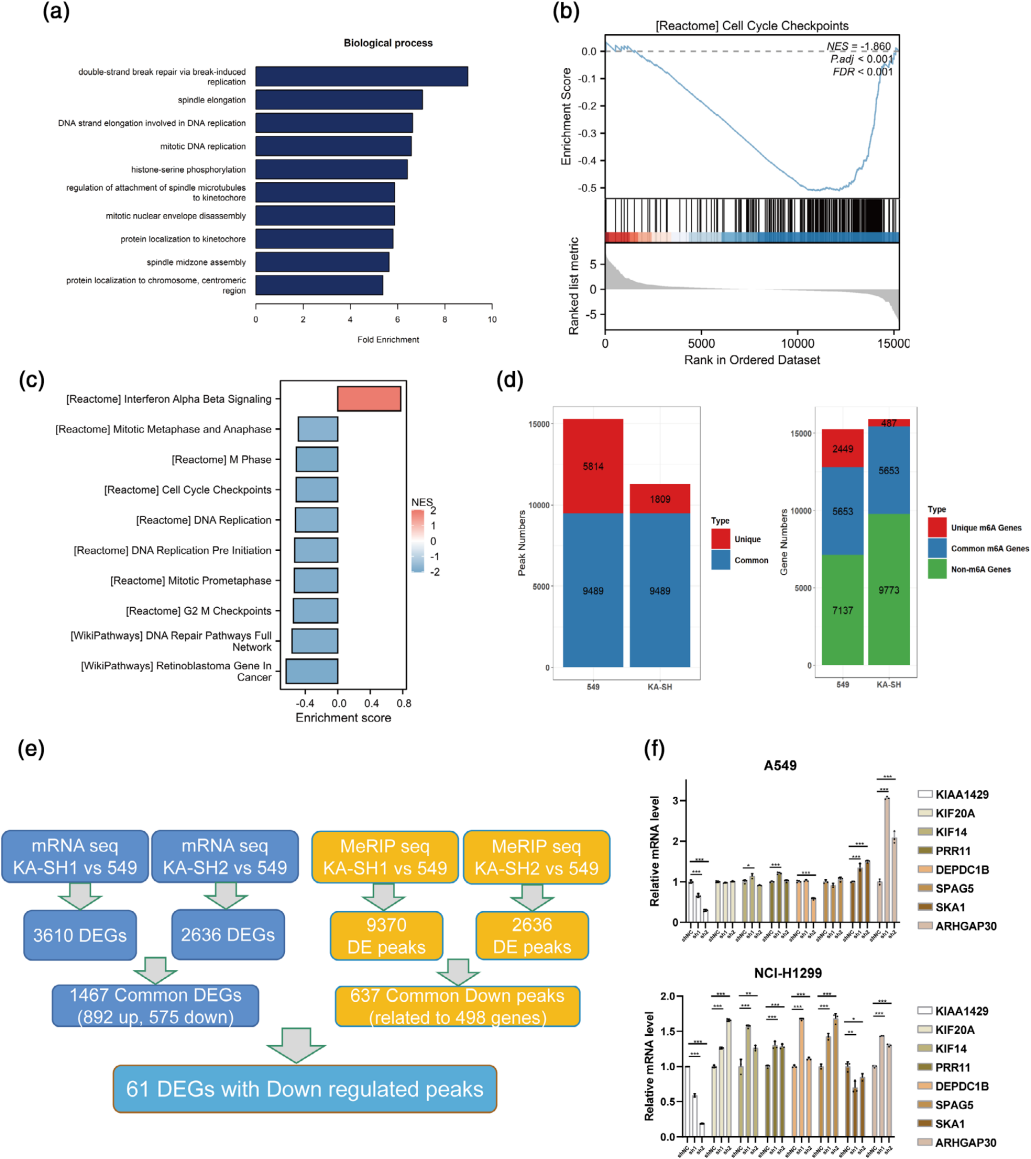
KIAA1429 affected the overall m6A modification level. (a) Biological process (BP) analysis of genes showing altered expression upon KIAA1429 inhibition. (b) The GSEA analysis revealed an increase in gene clusters crucial for cell cycle checkpoints following the KIAA1429 knockdown. (c) Gene ontology (GO) analysis. (d) The peak number was reduced after knockdown of KIAA1429 in MeRIP‐seq. (e) Combined RNA‐seq and MeRIP‐seq analysis for downstream differential genes. (f) Changes in gene mRNA expression were specifically verified through quantitative reverse transcription‐polymerase chain reaction (qRT‐PCR) in cells with reduced KIAA1429. Three independent experiments were performed. Data are shown as the mean ± SD. *p < 0.05; **p < 0.01; ***p < 0.001; ****p < 0.0001, *ns* not significant.

We delved into the biological processes these genes are involved and discovered enrichment in pathways related to double‐strand break repair via break‐induced replication, spindle elongation, and mitotic DNA replication (Figure 3a). Evidence from gene set enrichment analysis (GSEA) indicated an inverse relationship with cell cycle checkpoints (Figure 3b), while gene ontology (GO) analysis pointed to enrichment in M phase, cell cycle checkpoints, and DNA replication processes (Figure 3c).

MeRIP‐seq analysis post‐KIAA1429 knockdown showed a decrease in the number of m6A peaks and a reduced count of methylated genes (Figure 3d). Using a stringent threshold of FC >3 and *p*‐value <0.05, we identified differential methylation peaks: 9370 in KA‐SH1 and 2636 in KA‐SH2. A significant overlap in these peaks, greater than 1 nucleotide, was noted, highlighting 637 common methylation downregulated peaks corresponding to 498 genes (Figure 3e). Finally, we employed qRT‐PCR to validate the changes in mRNA expression as identified by the common biosignature analysis (Figure 3f). Our rigorous multidimensional analysis delineates the extensive regulatory network influenced by KIAA1429 and its significant epigenetic impact on gene expression in LUAD.

### KIAA1429 promotes LUAD cell proliferation and migration by inhibiting ARHGAP30 expression

**FIGURE 4:**
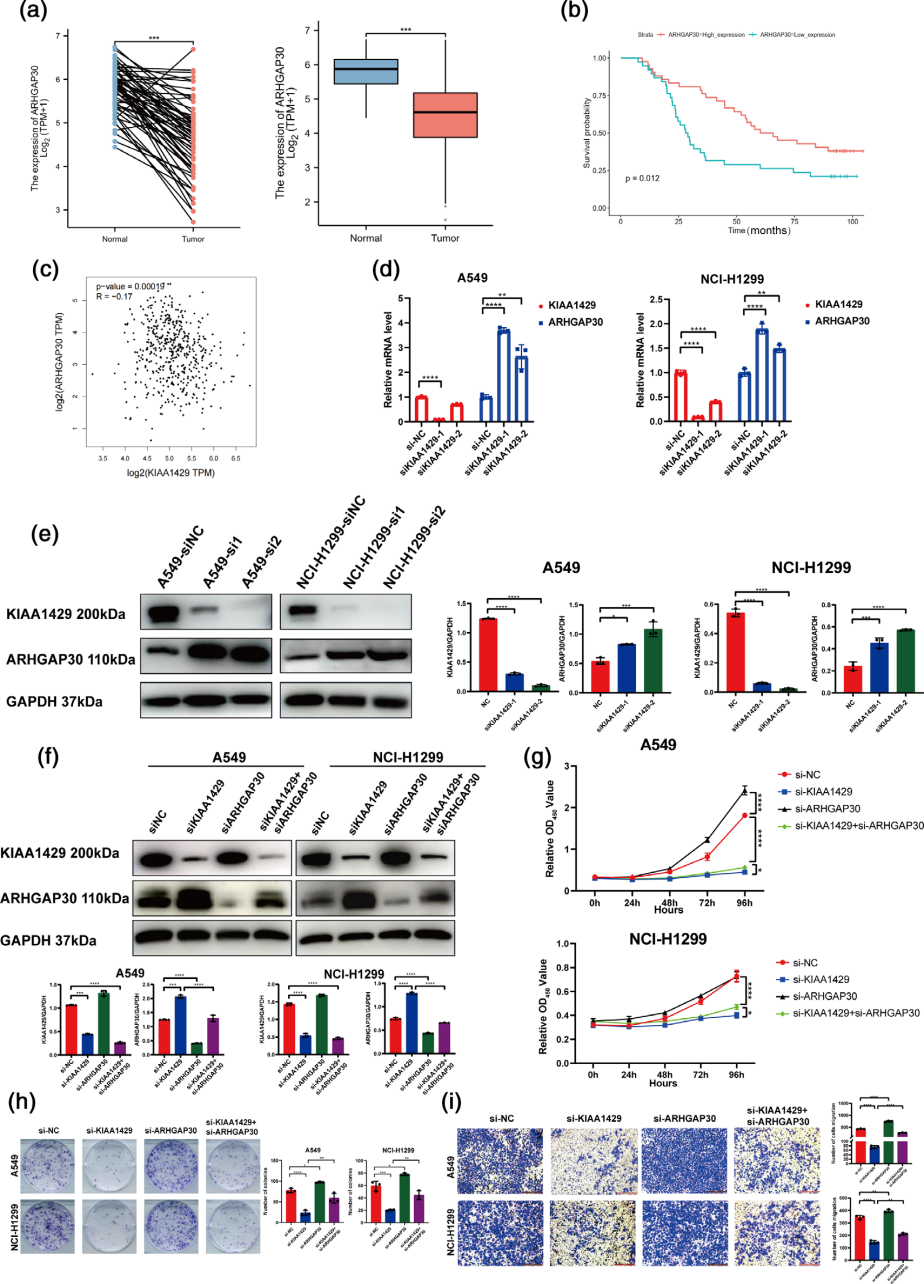
KIAA1429 promotes lung adenocarcinoma (LUAD) cell proliferation and migration by inhibiting ARHGAP30 expression. (a) The TCGA database was utilized to examine ARHGAP30 expression in LUAD tissues. (b) According to TCGA LUAD data, reduced expression of ARHGAP30 suggests lower survival rates in contrast to its elevated expression levels. (c) The correlation examination of LUAD tissues from TCGA databases revealed a slight inverse relationship between KIAA1429 and ARHGAP30. (d) ARHGAP30 mRNA levels were measured using qRT‐PCR after KIAA1429 suppression. (e) Post the silencing of KIAA1429, western blot tests revealed the presence of KIAA1429 and ARHGAP30, in contrast to the control groups in A549 and NCI‐H1299 cells. (f) Western blot assays detected the protein levels of ARHGAP30 in A549 and NCI‐H1299 cells after relative treatment. (g) Cell counting kit‐8 (CCK8) assays were conducted to investigate the proliferation of A549 and NCI‐H1299 cells after relative treatment. (h) Assays for colony formation were conducted in A549 and NCI‐H1299 cells after relative treatment. (i) The migratory capacities of A549 and NCI‐H1299 cells were assessed using transwell assays after relative treatment. Scale bar, 200 μm. Three independent experiments were performed. Data are shown as the mean ± SD. *p < 0.05; **p < 0.01; ***p < 0.001; ****p < 0.0001, *ns* not significant.

Within the spectrum of KIAA1429's potential targets, our attention was drawn to the ARHGAP30 gene, which displayed marked upregulation following the knockdown of KIAA1429. Earlier studies have identified this gene as a suppressor of tumors, playing a role in multiple cancer‐causing routes. A review of the TCGA database revealed reduced expression of ARHGAP30 in LUAD tissues relative to noncancerous tissues (Figure 4a). Furthermore, low ARHGAP30 expression correlated with a poorer prognosis in LUAD patients (Figure 4b).

An inverse relationship between KIAA1429 and ARHGAP30 expression was also evident in our analysis of the TCGA database (Figure 4c). The suppression of KIAA1429 resulted in increased ARHGAP30 expression at both mRNA and protein levels (Figure 4d,e). More importantly, when ARHGAP30 was knocked down on top of knockdown of KIAA1429, ARHGAP30 protein levels were partially upregulated in this case compared to knockdown of ARHGAP30 alone (Figure 4f). Additionally, the inhibition of cell growth and migration that was initially observed with KIAA1429 knockdown was partially negated by the subsequent downregulation of ARHGAP30 (Figure 4g–i).

These results suggest that ARHGAP30 may act downstream of KIAA1429 and play a significant role in the tumor‐suppressive pathways affected by KIAA1429 in LUAD, offering potential insights into the molecular interplay that could be leveraged for therapeutic strategies in LUAD.

### KIAA1429 affects the expression of ARHGAP30 by regulating m6A level and RNA stability

**FIGURE 5:**
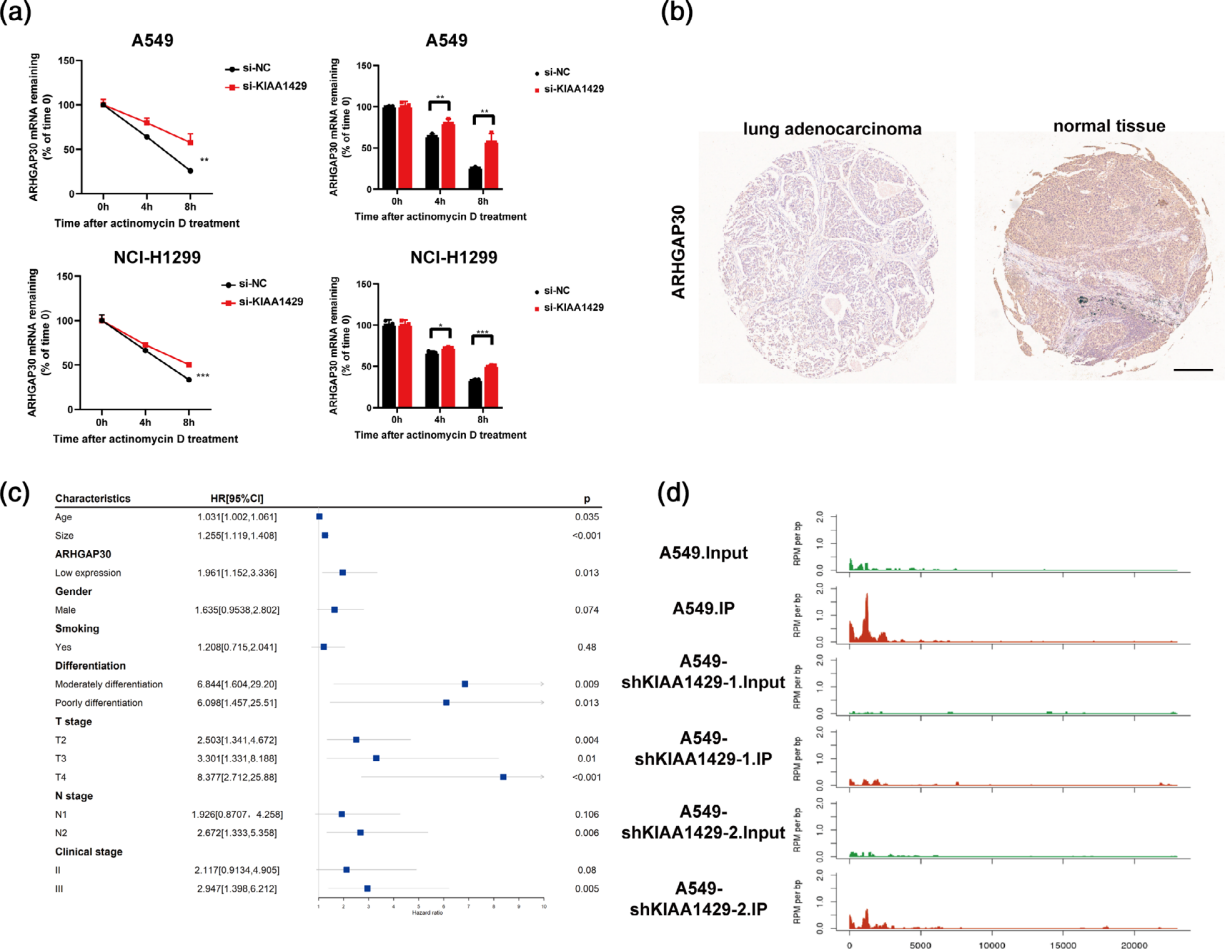
KIAA1429 affects the expression of ARHGAP30 by regulating m6A level and RNA stability. (a) Stability of ARHGAP30 mRNA in KIAA1429 knockdown cells. (b) Immunohistochemistry of the ARHGAP30 gene in lung adenocarcinomas and normal tissue. Scale bar, 500 μm. (c) Univariate meta‐regression for different subgroup analyses. (d) There was a decrease in the count of m6A peaks within the ARHGAP30 gene area after knockdown of KIAA1429 in MeRIP‐seq sequencing. Three independent experiments were performed. Data are shown as the mean ± SD. *p < 0.05; **p < 0.01; ***p < 0.001; ****p < 0.0001, *ns* not significant.

Post suppression of KIAA1429, there was a notable enhancement in the mRNA stability of ARHGAP30 (Figure 5a). This finding aligns with our immunohistochemical analysis on tumor tissues, which also revealed a low expression level of ARHGAP30 (Figure 5b). In a clinical cohort analysis (Figure 5c), the hazard ratio (HR) for the low‐expression group of ARHGAP30 was 1.961 with a *p*‐value of 0.013. This suggests that ARHGAP30 is expressed at low levels in LUAD patients, a finding of statistical significance.

Additionally, in the T stage analysis, the HR for the T4 stage was 8.377, indicating that low ARHGAP30 expression at the T4 stage minimally reduces the risk of recurrence compared to high expression levels. Notably, the horizontal line segment in this analysis does not intersect with the null line, further confirming the statistical significance of these findings.

Moreover, MeRIP‐seq results revealed a decrease in the methylation level of the ARHGAP30 gene region following the knockdown of KIAA1429 (Figure 5d). This decrease in methylation correlates with the observed increase in ARHGAP30 mRNA stability and expression, reinforcing the regulatory role of KIAA1429 in the epigenetic modification of ARHGAP30 in LUAD. This ensemble of data underscores the intricate molecular interplay between KIAA1429 and ARHGAP30, highlighting their potential as critical targets in the study and treatment of LUAD.

### KIAA1429 affects LUAD proliferation through the PI3K/AKT signaling pathway by regulating the expression of ARHGAP30

**FIGURE 6:**
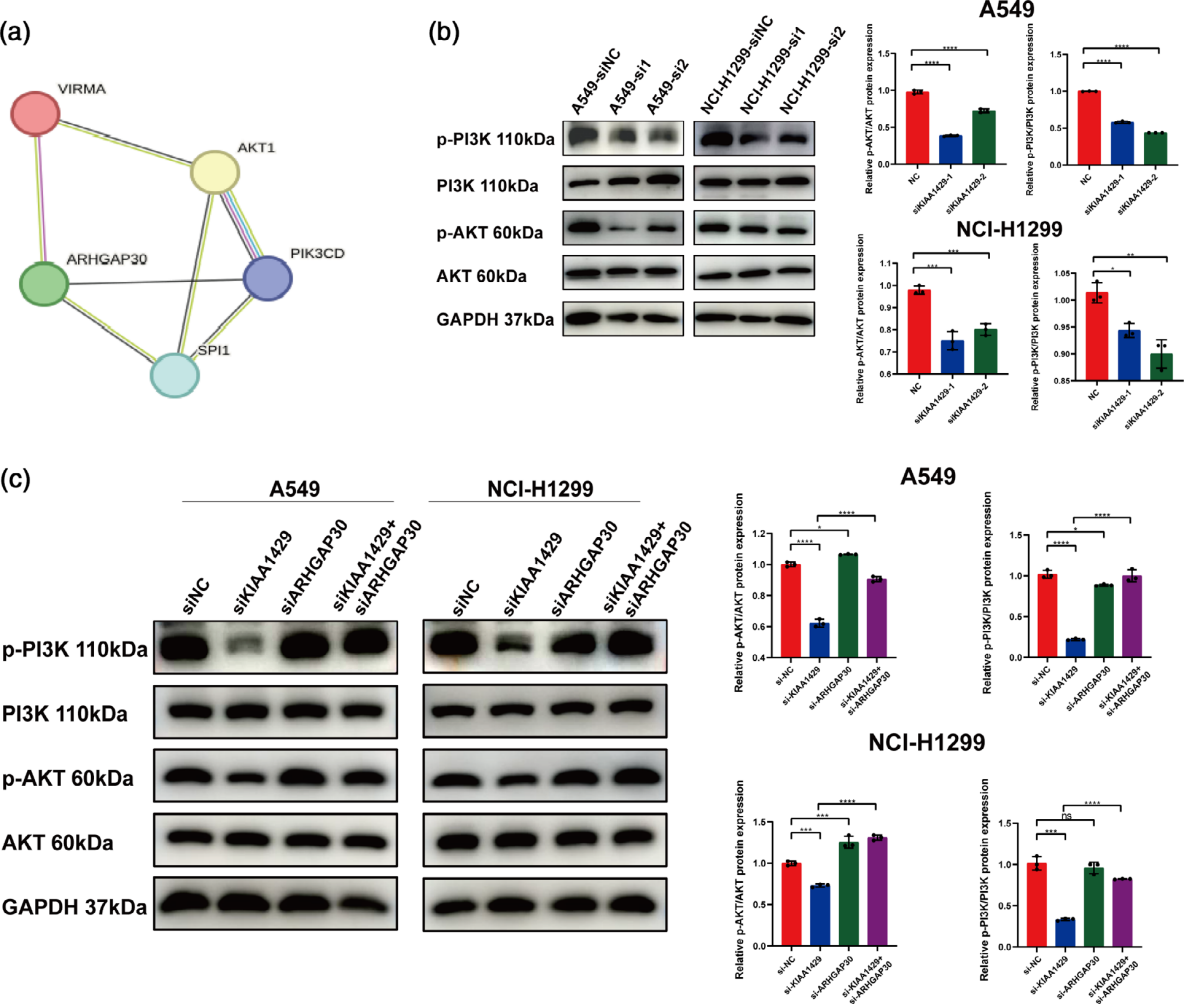
KIAA1429 affects lung adenocarcinoma proliferation through the PI3K/AKT signaling pathway by regulating the expression of ARHGAP30. (a) According to the STRING database, the interplay between ARHGAP30 and the PI3K/AKT pathway was forecasted. (b) Following transfection with siNC and siKIAA1429 in A549 and NCI‐H1299 cells, western blotting was used to detect the expression of p‐PI3K, PI3K, p‐AKT, and AKT. (c) After transfection with siNC, siKIAA1429 and siARHGAP30 in A549 and NCI‐H1299 cells, the expression of p‐PI3K, PI3K, p‐AKT, and AKT were identified using western blotting. Three independent experiments were performed. Data are shown as the mean ± SD. *p < 0.05; **p < 0.01; ***p < 0.001; ****p < 0.0001, *ns* not significant.

Utilizing the STRING database (https://cn.string-db.org/), investigations revealed the interaction of ARHGAP30 with the PI3K/AKT/mTOR signaling pathway (Figure 6a). Considering earlier research showing ARHGAP30's tumor‐inhibiting function in LUAD by hindering LUAD cell growth, we aimed to explore whether KIAA1429 influences the PI3K/AKT signaling pathway via its regulatory effect on ARHGAP30, thereby impacting LUAD proliferation and metastasis.

During our tests involving A549 and NCI‐H1299 cells, inhibiting KIAA1429 led to a notable reduction in the levels of activated PI3K (p‐PI3K) and activated AKT (p‐AKT) (Figure 6b). This finding suggests a potential regulatory mechanism by which KIAA1429 might modulate the PI3K/AKT pathway. To further probe this relationship, we performed a subsequent knockdown of ARHGAP30 on the background of KIAA1429 knockdown. This dual knockdown led to a partial reversal in the expression of p‐PI3K and p‐AKT (Figure 6c).

The findings together reveal that KIAA1429 enhances the control of the PI3K/AKT pathway in LUAD cells by decreasing ARHGAP30 mRNA levels, thus encouraging LUAD's metastasis and growth. This intricate molecular interplay between KIAA1429, ARHGAP30, and the PI3K/AKT signaling pathway offers significant insights into the mechanistic pathways driving LUAD progression, highlighting potential targets for therapeutic intervention in this form of lung cancer.

## DISCUSSION

The study concentrated on KIAA1429's function in lung adenocarcinoma (LUAD), disclosing its elevated presence in LUAD tissues and its link to unfavorable outcomes, as evidenced by examining the TCGA dataset. The studies conducted in vitro demonstrated that KIAA1429 markedly affects cell growth and migration in LUAD, and also alters the total levels of N6‐methyladenosine (m6A) modification in these cells. Significantly, our findings suggest that KIAA1429 influences the steadiness of ARHGAP30 mRNA in a way reliant on m6A, a process that drives the evolution and advancement of LUAD tumors.

Our study contributes valuable insights into the molecular mechanisms of LUAD tumorigenesis, particularly highlighting the pivotal role of KIAA1429 and its potential as a therapeutic target in LUAD treatment. Initial research suggests that RNA methylation is an important regulatory part of epigenetics and identified m6A as the key to RNA methylation.^16^ RNA methyltransferases, KIAA1429 among them, function as the “writers” for m6A, significantly contributing to these unconventional alterations.^17^, ^18^ Earlier studies have revealed atypical expression of RNA methyltransferases in different cancerous tumors,^13^ yet their downstream regulatory mechanisms have seldom been reported.

KIAA1429 serves as an m6A “writers” playing a crucial role in the emergence and progression of tumors. This process engages fundamental catalytic elements including METTL3, METTL14, and WTAP to modulate the m6A modification.^13^ Furthermore, KIAA1429 influences MUC3A expression and resistance of NSCLC to gefitinib by targeting HOXA1 3'UTR.^19^

The results of our study reveal that KIAA1429 influences the levels of m6A in LUAD cells. Further RNA‐seq and MeRIP‐seq studies have revealed the pivotal role of KIAA1429 in regulating ARHGAP30. ARHGAP30, a Rho‐specific Rho GAP,^20^ has been reported to have tumor‐suppressive properties. The expression of ARHGAP30 is frequently reduced in various tumors, such as LUAD, especially because of changes in DNA methylation in its promoter area.^21^ Additionally, ARHGAP30 plays a crucial role in the acetylation of p53 and its activation amid DNA damage stress in colorectal cancer (CRC), and its expression levels are linked to survival rates in CRC patients.^22^ The study revealed that RNA methylation modification (m6A) acts as an epigenetic indicator, inhibiting ARHGAP30 expression in LUAD.

Within LUAD tissues, ARHGAP30, a tumor suppressor, shows a substantial decrease and a negative correlation with KIAA1429 expression, suggesting KIAA1429‐triggered m6A alterations directly affect ARHGAP30. The finding highlights the expected cancer‐causing impacts of KIAA1429 in LUAD evolution, influencing subsequent target genes via epigenetic RNA, adding a new dimension to the epigenetic transformation seen in LUAD.

In conclusion, our study provides a thorough understanding of KIAA1429's role in the progression of LUAD. The research underscores a high occurrence of KIAA1429 in LUAD tissues, showing a strong link between its increased expression and a poorer prognosis for patients. In vitro studies conducted showed that KIAA1429 influences cell growth and migration, playing a pivotal role in m6A's modification of RNA, which has an impact on the development and progression of LUAD tumors. An essential element of our research lies in pinpointing ARHGAP30 as a consequent target of KIAA1429, controlled through modulation of mRNA stability dependent on m6A. The regulatory mechanism of KIAA1429 suggests it might be a key carcinogenic element in LUAD, providing crucial understanding for subsequent treatment approaches. The insights from our study illuminate the intricate molecular aspects of LUAD and also enhance the existing knowledge concerning RNA alterations and their impact on cancer biology, creating fresh paths for precision treatments in LUAD.

## AUTHOR CONTRIBUTIONS

Wei Guo: Conceptualization, funding acquisition, data curation, methodology, project administration, investigation, resources, writing—original draft, writing—review and editing. Tan Wang: Investigation, data curation, methodology, writing—original draft, writing—review and editing. Qilin Huai: Investigation, resources, writing—review and editing. Xiaobing Wang: Funding acquisition, project administration, resources and supervision. Jie He: Project administration, resources and supervision.

## CONFLICT OF INTEREST STATEMENT

The authors declare no competing interests in relation to this study.

## FUNDING INFORMATION

This study was supported by the National Key Research and Development Program of China (2021YFC2500900), the National Natural Science Foundation of China (82002451, 82273129), Beijing Nova Program (20230484267), the CAMS Initiative for Innovative Medicine (2021‐I2M‐1‐015, 2023‐I2M‐C&T‐B‐078), Central Health Research Key Projects (2022ZD17), and Research Project of the Institute (LC2019L01).

## Supporting information


**Table S1.** The detailed clinical characteristics of the 80 LUAD patients.

**Table S2.** The sequences of primers, siRNAs and shRNAs.

**Table S3.** Table of sequencing results of mRNA before and after knockdown of KIAA1429.

## Data Availability

The raw data supporting the findings of this study are available from the corresponding author upon reasonable request.
